# Mental State Inferences Abilities Contribution to Verbal Irony Comprehension in Older Adults with Mild Cognitive Impairment

**DOI:** 10.1155/2015/685613

**Published:** 2015-06-25

**Authors:** G. Gaudreau, L. Monetta, J. Macoir, S. Poulin, R. Jr. Laforce, C. Hudon

**Affiliations:** ^1^École de Psychologie, Université Laval, Québec, QC, Canada G1V 0A6; ^2^Centre de Recherche de l'Institut Universitaire en Santé Mentale de Québec, Québec, QC, Canada G1J 2G3; ^3^Département de Réadaptation, Université Laval, Québec, QC, Canada G1V 0A6; ^4^Clinique Interdisciplinaire de Mémoire, Département des Sciences Neurologiques, CHU de Québec, Québec, QC, Canada G1J 1Z4

## Abstract

*Objective*. The present study examined mentalizing capacities as well as the relative implication of mentalizing in the comprehension of ironic and sincere assertions among 30 older adults with mild cognitive impairment (MCI) and 30 healthy control (HC) subjects. *Method*. Subjects were administered a task evaluating mentalizing by means of short stories. A verbal irony comprehension task, in which participants had to identify ironic or sincere statements within short stories, was also administered; the design of the task allowed uniform implication of mentalizing across the conditions. *Results*. Findings indicated that participants with MCI have second-order mentalizing difficulties compared to HC subjects. Moreover, MCI participants were impaired compared to the HC group in identifying ironic or sincere stories, both requiring mental inference capacities. *Conclusion*. This study suggests that, in individuals with MCI, difficulties in the comprehension of ironic and sincere assertions are closely related to second-order mentalizing deficits. These findings support previous data suggesting a strong relationship between irony comprehension and mentalizing.

## 1. Introduction

Given the aging of the population, the prevalence of dementia such as Alzheimer's disease (AD) is increasing. It is now largely accepted that early identification of AD is crucial. An important scientific and clinical challenge is to clarify the neuropsychological profile of individuals with mild cognitive impairment (MCI) to assist the identification of early AD-related cognitive deficits. The concept of MCI corresponds to the prodromal stage of AD in several, if not most, older adults (for reviews on MCI, see [1, 2]). Older adults with MCI show a cognitive decline (typically involving memory) greater than what would be expected in an individual given his/her age and education, without significant functional impairment [1].

Among the neuropsychological characteristics of individuals with MCI, mentalizing deficits have already been documented [3–5]. Mentalizing refers to the capacity to infer mental states, or make social inferences, about the mental state of others [6]. More specifically, mentalizing is the ability to metarepresent mental states and to refer to these metarepresentations in order to predict and understand the behavior of oneself or others [7]. Previous authors have indicated that mentalizing capacities are required for many other cognitive processes such as an adequate comprehension of verbal irony [8–12].

Verbal irony comprehension is generally defined as a linguistic process used to express information which is directly, or indirectly, opposed to its literal interpretation [13]. Recent studies evidenced verbal irony comprehension difficulties in older adults with MCI [14, 15]. However, those studies did not systematically control for mentalizing contribution among task's conditions. One could thus wonder whether the verbal irony deficits found in MCI simply reflect mental state inference problems in this population. Controlling for mental state inferences across task condition is necessary to comprehend the origin of verbal irony comprehension deficits.

The main objectives of this study are twofold: (a) to corroborate previous results indicating the presence of mentalizing deficits in older adults with MCI and (b) to examine the implication of mentalizing in the comprehension of verbal assertions based on mental state inferences (i.e., ironic and sincere assertions) in MCI. To this end, participants were administered two different tasks, one evaluating mentalizing capacities and the other verbal irony comprehension by means of short stories. The mentalizing task assessed the integrity of first- (i.e., inferring somebody else's mental state) and second-order (i.e., inferring what somebody might think about another's mental state) mentalizing, as well as nonsocial reasoning (i.e., inference of facts or events that implicate no human beings). The verbal irony task assessed the interpretation of ironic and sincere scenarios. Considering existing bodies of evidence of second-order mentalizing deficits in individuals with MCI [3, 5, 16], it is hypothesized that participants with MCI in the present study will show impaired performance in the mentalizing task. With respect to verbal irony comprehension, it is predicted that interpretation of both ironic and sincere story types will be impaired in MCI compared to healthy control participants. This pattern of results will suggest that mentalizing abilities are needed for the comprehension of both types of stories or mental state inferences (ironic and sincere). Finally, given that mentalizing and verbal irony comprehension are related [8–12], exploratory analyses were conducted to examine the association between performance of participants in the mentalizing and verbal irony comprehension tasks. Moreover, since mentalizing has been shown to correlate with episodic memory and executive functions capacities [17–21] and given that these cognitive functions are impaired in MCI, a last set of exploratory analyses has been carried out to verify these associations in the participants of this study.

## 2. Method

### 2.1. Participants

Participants included 30 older adults with MCI (age range: 60–83 years) and 30 healthy control (HC) subjects (age range: 55–82 years). Participants with MCI were identified and referred to the research team by experienced geriatricians, neurologists, or general practitioners, or through advertisements displayed in different clinics. All MCI participants met the clinical core criteria of Albert et al. [1]. All MCI participants had episodic memory impairment and this was the only cognitive impairment in six individuals. The remaining 24 subjects had additionally one or more nonmemory impairments (e.g., executive deficits). The diagnosis of MCI was confirmed based on a battery of clinical and neuropsychological tests and each case was discussed by a team of clinicians in order to reach a consensus regarding the status of participants. No participant with MCI met AD criteria. As regards participants of the HC group, they were recruited in the community on a voluntary basis. They were all in good physical and mental health at the time of the study and they all scored above −1 SD on standardized neuropsychological tests based on norms stratified for age and education. All participants spoke French as their primary language.

For all participants, exclusion criteria were as follows: (a) history of traumatic brain injury; (b) history of stroke or other cerebrovascular disorders; (c) history of delirium (in the last six months); (d) formal intracranial surgery; (e) history of neurological disorder of cerebral origin or associated with another dementia state (e.g., parkinsonism, multiple sclerosis, etc.); (f) history of encephalitis or bacterial meningitis; (g) unstable metabolic or medical condition (e.g., uncontrolled diabetes or hypothyroidism); (h) history or current diagnosis of a psychiatric illness or dementia according to the DSM-IV [22]; (i) oncological treatment in the past 12 months; (j) alcoholism/drug addiction according to the DSM-IV [22]; (k) general anaesthesia in the last 6 months; (l) uncorrected vision or hearing problems; (m) use of experimental medication (i.e., medication that has not yet received approval from governmental regulatory authorities). Exclusion criteria were verified based on participants' report and confirmed with a close relative. In some cases, participants' medical file was also examined.

An informed consent form was signed by each participant at the beginning of the first assessment session. This study was approved by the Ethics Research Board of the* Institut Universitaire en Santé Mentale de Québec*.

### 2.2. Materials

#### 2.2.1. Neuropsychological Battery

Participants were administered a comprehensive battery of clinical and neuropsychological tests in order to verify inclusion/exclusion criteria and to characterize their cognitive and clinical status. The battery included instruments to assess depressive symptoms (Geriatric Depression Scale [23]), functional autonomy (Alzheimer's Disease Cooperative Study-Activity of Daily Living [24]), cognitive complaint (questionnaire de plainte cognitive (Cognitive Complaint Questionnaire) [25]), general cognitive status (Dementia Rating Scale 2 [26]; Montreal Cognitive Assesment [27]), executive functions (Stroop Color-Word Test and Trail Making Test [28]; letter-number sequencing [29]), visuoconstructive abilities (Rey Complex Figure Task [30]), visuoperception (Birmingham Object Recognition Battery Size-Match Task [31]), confrontation naming (15-Item Boston Naming Test [32]), lexical (T-N-P) and semantic (animals) verbal fluency [33], semantic memory (Pyramids and Palm Trees Test [34]), and episodic memory (rappel libre/rappel indicé à 16 items (Free Recall/Cued Recall 16-Item Test) [35]).

#### 2.2.2. Mentalizing Abilities

First- and second-order mentalizing abilities were evaluated using the* Combined Stories Test*, a task developed and validated by Achim et al. [36]. A total of 30 short stories, including one practice trial, were presented to participants. For each story, a maximum of three questions were asked, evaluating either second-order mentalizing (26 questions), first-order mentalizing (3 questions), nonsocial reasoning (i.e., inference of facts or events that implicate no human beings; 6 questions), or attention/memory (30 questions, one for each scenario). First- and second-order mentalizing, as well as nonsocial reasoning questions, were scored as 2 (complete answer), 1 (incomplete answer), or 0 (incorrect or unrelated answer). Responses for attention/memory questions, which are scored as 1 (correct) or 0 (incorrect), were asked at the end of each scenario. The scenarios of the Combined Stories Test were presented on a laptop computer screen using the Microsoft Office PowerPoint software. Questions were presented verbally by the examiner. The principal variable of interest in this task was the score on second-order mentalizing questions. Scores on nonsocial reasoning and first-order mentalizing questions were also analyzed but these scores were used for comparison purposes to help interpreting any mentalizing deficit. Responses to attention/memory questions were not analyzed because these items were simply aimed at ensuring literal understanding of each story. Attention/memory questions were correctly answered 94.6% of the time in MCI participants and 95.0% of the time in the HC group. Since these ratios were almost identical between groups and because they were likely representative of normal error margin, every story was analyzed regardless of responses on attention/memory questions. This is consistent with the procedure of Achim et al. [36].

#### 2.2.3. Verbal Irony Comprehension

The* Short Scenario Irony Comprehension Task* (SSICT), a French task of verbal irony comprehension adapted from Eviatar and Just [37], was used. Adaptation and translation of the task were made by the second author (LM). The SSICT was validated in a group of 75 healthy participants. This validation study allowed selecting the scenarios successfully comprehended by 80% of the participants [38]. Stimuli of the SSICT were presented on a laptop computer screen using the E-Prime 2 software (Psychology Software Tools, Sharpsburg, PA).

The SSICT consists in written presentation of 14 short stories describing everyday life situations, each including four assertions (for a sample of the task see the following). Sample story in French adapted from Eviatar and Just [37] is as follows.

*Le Placotage (The Chatting)*

 Julie est bavarde et elle ne peut s'empêcher de tout commenter. (*Julie is a talkative person and she cannot help commenting on anything*.)
 Son ami Robert a l'habitude de l'entendre parler sans cesse et cela le fait bien rire. (*Her friend Robert is used to hear her talking incessantly and this fact amuses him*.)
 Un jour, ils se rencontrent pour déjeuner, mais Julie est fatiguée et elle ne parle presque pas. (*One day they meet for breakfast, but Julie is tired and she hardly speaks.*)

*Sincere Statement*. Je dois lire dans tes pensées ce matin, dit Robert. (*I have to read your mind this morning, says Robert.*)

*Ironic Statement*. Tu es en forme pour un débat, dit Robert. (*You are fit for a debate, said Robert.*)

*Descriptive Statement*. Le déjeuner comporte du lait et des céréales. (*A breakfast includes milk and cereals.*)

The first sentence always presents the general context of the scenario, whereas the second phrase describes the specific context. The third statement presents a triggering event. Finally, the fourth sentence is the one that has to be evaluated as being either (a) sincere, (b) ironic, or (c) descriptive. Of the 14 scenarios, five describe a sincere situation, five are ironic, and four are descriptive. The fourth statement of the* sincere* and* ironic* scenarios is considered “social” (i.e., it refers to an interaction between two protagonists), while the last statement in* descriptive* scenarios is “nonsocial” (i.e., it describes neutrally a physical element of the story and does not implicate any protagonist). The presence of descriptive scenarios in the task is aimed at minimizing the response bias that can occur when tasks involve dichotomous answers (e.g., ironic versus sincere). To summarize, except for the very last sentence, which varies depending on the story type, all scenarios comprise the interaction between two protagonists and present one triggering event. Moreover, the scenarios are all relatively short (approximately 45 words) in order to minimize the contribution of nonlanguage (e.g., memory and executive functions) cognitive functions.

To correctly interpret both sincere and ironic scenarios, participants must infer or metarepresent mental states. It is assumed that the “mentalizing load” is comparable between sincere and ironic scenarios since, in both cases, the interaction between two protagonists is depicted. Moreover, for both sincere and ironic stories, participants have to infer a mental state in one protagonist by taking into account the context described in the first three literal assertions. The variable of interest in the SSICT is the correct interpretation (i.e., response accuracy) of the fourth sentence, which is scored as 1 or 0.

### 2.3. Procedure

Participants were assessed individually over three sessions. The first session included the signature of the informed consent form and the administration of the clinical and neuropsychological tests or questionnaires; this session lasted approximately two hours. Mentalizing abilities (Combined Stories Test) and verbal irony comprehension (SSICT) were assessed during the second and third sessions, in a counterbalanced order. These sessions lasted approximately 30 to 45 minutes each.

#### 2.3.1. Mentalizing Abilities

Participants sat at about 1 m from the computer screen and were asked to read the stories out loud. When finished, the experimenter asked questions (first- and second-order mentalizing, nonsocial reasoning, and attention/memory) and wrote down the participant's answer on the response sheet.

#### 2.3.2. Verbal Irony Comprehension

Participants sat at about 1 m from the computer screen. They were asked to read out loud the task instructions, which appeared on the computer screen. They were then asked to read the written stories out loud in a self-paced manner. When the first two sentences of a story were read, participants pressed the “Enter” button in order to see and read sentences 3 and 4 (the first two sentences remained on the screen at this stage). Following reading of the fourth sentence, subjects were asked whether the story was (1) sincere, (2) ironic, or (3) descriptive. They had to answer by pressing the corresponding number on a keypad placed in front of them. Stories were counterbalanced in a random order to control for a possible sequence effect.

### 2.4. Statistical Analyses

#### 2.4.1. Demographic and Clinical Data

Except for sex distribution in the groups, which was analysed using Pearson Chi-square test, Student's *t*-tests were used to compare MCI and HC participants on demographic and clinical data. These analyses were performed (a) to ensure equivalence of the groups with respect to age, sex, and education and (b) to compare the clinical and neuropsychological profile of participants.

#### 2.4.2. Experimental Tasks

Exploratory analyses were first conducted to examine normal distribution assumptions required for inferential analyses. All variables of interest were normally distributed. Regarding the Combined Stories Test, proportions of correct responses were calculated and Student's *t*-tests were used to compare the groups on first- and second-order mentalizing, as well as nonsocial reasoning. For the SSICT, proportions of correct responses were calculated for each scenario and a mixed ANOVA Group (HC and MCI) X Story type (sincere and ironic) was used in order to compare group performance.

#### 2.4.3. Relationships between Variables

Pearson's correlations were used to assess the association between the key variables of the experimental tasks (i.e., stories evaluating second-order mentalizing in the Combined Stories Test and stories evaluating comprehension of ironic and sincere scenarios in the SSICT). With regard to the SSICT, the relationship between results for ironic and sincere stories was also analyzed, to examine statistically the extent to which these two conditions rely on similar cognitive processes. Moreover, Pearson's correlations were performed in order to analyse associations between the score on variables of interest in the Combined Stories Test and the SSICT with measures of executive functions and episodic memory (mean score of immediate free and total recall from the Free Recall/Cued Recall 16-Item Test [RL/RI-16]). In order to limit the number of analyses, a composite score was computed for executive functions, including measures of manipulation of information in working memory (score on the WAIS-III letter-number sequencing task), controlled inhibition (time and total number of errors on the third condition of the D-KEFS Color-Word Interference Test), and mental flexibility (time and total number of errors on the fourth conditions of the D-KEFS Color-Word Interference and the Trail Making Tests). *Z*-scores were first computed using the mean and standard deviations of the control group for each measure included in the composite score. Then, the derived *Z*-scores were averaged in order to quantify the difference in executive functions in MCI participants compared to HC participants. The mean executive composite score for the MCI group was −1.92 (SD = 2.52).

For all analyses, an alpha level of .05 was used.

## 3. Results

### 3.1. Demographic and Clinical Data

Table 1 presents the demographic and clinical characteristics of the MCI and HC groups. The groups were comparable for age, years of education, and sex distribution. Participants with MCI had a greater number of depressive symptoms, a poorer general cognitive status, and more extensive cognitive complaints compared to the HC group. Regarding depressive symptoms, one should note that they were not associated with variable of interest in this study. Thus, they were not added as covariates in the main analyses. The score on the functional autonomy questionnaire was significantly lower in the MCI compared to the HC group, but as per inclusion criteria, MCI participants were not deemed to have clinically significant functional impairment. Moreover, the MCI group showed worse performance than HC participants in all cognitive domains, except for the number of errors in the D-KEFS Trail Making Test (executive function).

### 3.2. Mentalizing Capacities

**Figure 1:**
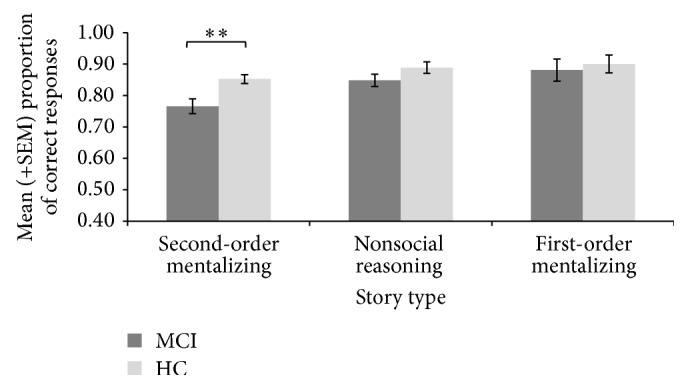
Mean (±SEM) proportions of correct scores on second-order mentalizing, nonsocial reasoning, and first-order mentalizing of the Combined Stories Test. MCI = elderly persons with mild cognitive impairment; HC = healthy controls participants; SEM = standard error of the mean ^*∗∗*^ *p* < 0.01.

Two participants out of 30 were excluded from the MCI group because of missing data or withdrawal from the study. Thus, 28 MCI participants were included in the final analyses.  Figure 1 reports the mean (±SEM) total correct scores for first- and second-order mentalizing, as well as nonsocial reasoning of the Combined Stories Test. The analyses revealed that MCI participants had more difficulty than the HC group in correctly answering the second-order mentalizing questions, *t*(57) = −2.74, *p* = 0.003, and *d* = .98. However, the groups were comparable regarding their capacity to answer nonsocial reasoning, *t*(57) = −1.48, *p* = 0.145, and *d* = .16, and first-order mentalizing, *t*(57) = −0.51, *p* = 0.609, and *d* = .03, questions.

### 3.3. Verbal Irony Comprehension

**Figure 2:**
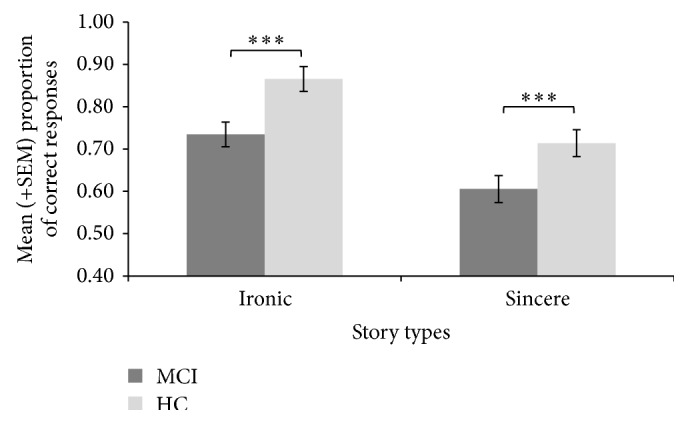
Mean proportions of correct responses (±SEM) for ironic and sincere stories of the Short Scenario Irony Comprehension Task. MCI = elderly persons with mild cognitive impairment; HC = healthy control participants; SEM = standard error of the mean ^*∗∗∗*^ *p* < 0.001.

Figure 2 illustrates the proportions of correct responses on sincere and ironic stories of the SSICT. The analysis indicated a significant main effect of the group,  *F*(1, 58) = 19.14, *p* < 0.001, and *η* ^2^ = .25, revealing that overall performance of MCI was worse than that of HC participants. There was also a main effect of story type, *F*(1, 58) = 11.96, *p* < 0.001, and *η* ^2^ = .17, indicating that ironic assertions were better interpreted by the participants compared to sincere ones. Finally, the interaction was not significant, *F*(1, 58) = .28, *p* = 0.756, and *η* ^2^ = .01.

### 3.4. Correlations between Variables

Overall, the second-order mentalizing score of the Combined Stories Test was significantly related to the scores on both ironic (*r*(59) = 0.40 and *p* = 0.001) and sincere (*r*(59) = 0.38 and *p* = 0.003) stories of the SSICT. A significant relationship was also found between ironic and sincere scores of the SSICT, *r*(61) = 0.32 and *p* = 0.011.

With respect to the SSICT, significant positive relationships were found between score in the sincere stories and mean scores of free (*r*(61) = 0.28 and *p* = 0.027) and total (*r*(61) = 0.40 and *p* = 0.001) immediate recall of the RL/RI-16. Significant positive correlations were also found between the composite executive score and sincere stories, *r*(59) = 0.36 and *p* = 0.006. A similar pattern of results was found for ironic stories as significant positive relationships were found between this variable and the mean scores of free (*r*(61) = 0.28 and *p* = 0.030) and total (*r*(61) = 0.26 and *p* = 0.047) immediate recall of the RL/RI-16. A significant positive relationship was also found between score in ironic stories and the composite executive score, *r*(59) = 0.30 and *p* = 0.021.

Finally, the second-order mentalizing score of the Combined Stories Test was significantly and positively related to the mean scores of free (*r*(58) = 0.43 and *p* = 0.001) and total (*r*(58) = 0.49 and *p* < 0.001) immediate recall of the RL/RI-16 and to the composite executive score, *r*(56) = 0.51 and *p* < 0.001.

## 4. Discussion

The main purposes of this study were (1) to confirm second-order mentalizing impairment in MCI and (2) to examine the relative implication of mental state inferences in the understanding of ironic and sincere assertions. With respect to second-order mentalizing abilities, results confirmed that MCI participants had significantly greater difficulties than their healthy counterparts. However, the groups were comparable regarding nonsocial reasoning and first-order mentalizing capacities. Regarding comprehension of ironic and sincere stories involving second-order mentalizing, participants with MCI were impaired relative to the HC group. That is, MCI participants had difficulty in correctly interpreting both ironic and sincere stories.

The present findings extend prior results published by Gaudreau et al. [14]. First, in Gaudreau et al. [14] the authors examined verbal irony comprehension in participants with MCI without considering mentalization abilities. Second, the tasks used in the present study address a limitation linked to the nature of materials used in Gaudreau et al. [14]. More precisely, the executive load has been considerably diminished in the tasks of the present study compared to those used in Gaudreau et al. [14]. Therefore, the mentalizing performance in the present study is less probably contaminated by executive difficulties in the participants.

Results of the Combined Stories Test [36] are in line with those of previous work showing second-order mentalizing deficits in older adults with MCI [3, 16]. The present study thus provides additional evidence that mental state inferences are impaired in prodromal AD. Our results are also in agreement with previous data showing that MCI participants have intact first-order mentalizing abilities [3, 5].

With respect to the SSICT, the MCI participants' difficulty in interpreting ironic written scenarios is in accord with previous results showing impaired verbal irony comprehension in this population [14, 15]. Interestingly, participants with MCI also had difficulty with questions relating to the sincere interpretation of the stories. As described earlier, sincere and ironic stories were manipulated to involve a similar social interaction between two protagonists. Therefore, participants had to rely on their mental state inferences abilities in order to correctly interpret both types of scenarios. Being unable to rely adequately on social inferences, individuals with MCI may have had difficulty in correctly inferring the mental state of the protagonist and thus in interpreting* both* the sincere and ironic connotation of the stories. This interpretation is supported by the fact that second-order mentalizing abilities, as measured by the Combined Stories Test, which were impaired in our MCI sample compared to the HC group, were also significantly and positively related to the comprehension of sincere and ironic assertions in the SSICT.

The significant relationship between second-order mentalizing and irony comprehension can also be explained by other factors. For instance, it could be that these cognitive processes are affected essentially to the same degree by the underlying neuropathology in MCI. Alternatively, the two measures could reflect to some extent a similar cognitive mechanism or process and thus impaired performance on one measure would be accompanied by impaired performance on the other. In fact, it is possible that the positive and significant association between performances in both experimental tasks derives from the underlying abilities in language and executive domains involved in both mental inferences abilities and verbal irony comprehension. Further studies will be necessary to verify these hypotheses.

The study of the relationships among the variables of interest brought interesting results, but the authors acknowledge that a better analysis strategy would have been to use linear regression instead of Pearson's correlation. Unfortunately, the sample size in this study was not large enough to use linear regression. That being said, the present results add to the literature by showing that the presence of episodic memory difficulties is significantly linked to poorer comprehension of ironic and sincere assertions as well as poorer second-order mentalizing capacities. Regarding executive functions, results are in line with previous data having demonstrated a relationship between these capacities and irony comprehension [11, 14, 39, 40]. More precisely, it appears that mental flexibility, controlled inhibition, and working memory are among the cognitive abilities implicated in the correct interpretation of an ironic, and even a sincere, assertion. This interpretation is coherent with the assumption that correct comprehension of an ironic commentary requires multiple “cognitive steps” (i.e., assessing both the literal and the inferred meaning of the assertion, while taking into account the context in which the phrase was pronounced) [13]. As for the relation found between executive functioning and second-order mentalizing, this result also corroborates previous work having demonstrated an interrelation between these constructs, both in healthy and brain-damaged populations [21, 41–45].

One interesting finding of the present study is the observation of a relationship between measures of episodic memory and the performance on both the SSICT and the Combined Stories Test. These results are consistent with the proposition of Moreau et al. [19] that episodic memory difficulties may be implicated in second-order mentalizing deficits. More precisely, episodic memory functioning may contribute to this nonliteral form of language comprehension. Consequently, the diverse cognitive deficits in individuals with MCI (i.e., second-order mentalizing, episodic memory, and executive functions) should be taken into account when evaluating verbal irony comprehension in this population.

One surprising result is that, in both MCI and HC groups, ironic assertions were better interpreted than sincere ones. An analysis of the error patterns indicated that sincere stories were often mistaken as being ironic, and vice versa. In MCI individuals, “ironic” responses consisted of 60% of the total number of errors for sincere scenarios, and this pattern was reversed for ironic scenarios (60% of the time, participants responded “sincere” instead of “ironic”). In the HC group, more than 68% of errors in sincere scenarios consisted of “ironic” responses. Again, the reverse pattern was found in ironic stories, these being identified as “sincere” 64% of the time. One explanation could rely on the fact that stories were read out loud by the participants and were not presented through a prerecorded and controlled voice. Thus, this administration procedure precluded the use of prosody to correctly differentiate sincere from ironic assertions. Prosody is known to be of considerable help when having to differentiate such statements [46].

Prior to this study, the adapted SSICT was validated in 75 young adults (mean age = 25.92; SD = 2.8) and results indicated that participants better understood sincere compared to ironic scenarios. The discrepancy between findings of the present study and those of the validation study suggests that the differentiation between irony and sincerity gets harder with aging. If so, this interpretation would be consistent with the fact that age impacts second-order mentalizing as measured by short stories, as well as text comprehension* per se* [47–49]. Aging is also known to affect information processing speed and executive functions [50] and the decline of these cognitive functions has been shown to impact second-order mentalizing capacities in healthy elders [5, 49, 51]. Henceforth, tasks generally used to evaluate verbal irony comprehension may need adaptations for the elderly (e.g., simplification and/or oral presentation of scenarios along with visual support).

The SSICT has strengths and weaknesses. On one hand, the use of mentalizing as a variable equally distributed in the sincere and ironic stories represents an important improvement in comparison with previous tasks. On the other hand, the standardization of mentalizing between ironic and sincere scenarios may have explained to some extent the overall deficit of MCI participants in the present study. In MCI, verbal irony comprehension could probably be better assessed by means of tasks using different modalities (e.g., videotaped scenarios or other ecologically oriented tasks). This question requires further investigation.

A limitation of the present study relates to the generalization of the results to all MCI cases, considering the rather small sample size. Because of the known heterogeneity of MCI, the sample in this study may not reflect all the characteristics that can be found in this population. Thus, mentalizing and verbal irony comprehension in MCI should be studied using larger samples. Moreover, longitudinal follow-up is warranted to determine to what extent these cognitive functions can help predict the dementia stage of AD in those individuals having MCI. In all, it is possible that the level of education influenced the performance of our participants, as a link between education and mentalizing was already demonstrated in the aging population [52]. This point would be interesting to address more comprehensively in further works on the subject.

Overall, this study provides additional evidence of second-order mentalizing impairment in older adults with MCI and suggests that mental state inferences deficits are associated with difficulties in the comprehension of ironic and sincere assertions in this population. It also brings support to the possible use of mentalization tests in the screening of individuals at risks of evolving towards AD, although this requires further investigation. Further studies should aim at improving the comprehension of the cognitive correlates of ironic and sincere assertions comprehension (e.g., executive functions and episodic memory) and their implication in second-order mentalizing capacities of individuals with MCI or healthy older adults. Such work will not only help identifying early the prodromal stage of AD but also help clarifying the theoretical grounds of the comprehension of verbal irony and sincerity.

## Figures and Tables

**Table 1:** Mean (SD) and significance levels of demographic and neuropsychological characteristics of participants.

Measures	MCI	HC	*t*-test	*p*
	(*n* = 30)	(*n* = 30)	*t* (1, 58)	
*Participants characteristics *				
Age (years)	73.9 (6.1)	71.9 (8.2)	1.2	0.230
Sex^a^	18F/12M	19F/11M	*χ* ^2^ = 0.071	0.791
Education (years)	13.6 (5.3)	14.2 (4.0)	−0.5	0.618
*Depressive symptoms *				
GDS (/30)	6.8 (4.6)	2.6 (2.6)	4.3^*∗∗*^	0.000
*Cognitive complaint *				
QPC (/10)	3.6 (2.3)	0.93 (1.4)	5.5^*∗∗*^	0.000
*General cognitive state *				
MoCA (/30)	23.0 (3.2)	26.5 (2.4)	−4.8^*∗∗*^	0.000
Dementia Rating Scale (/144)	134.0 (5.7)	139.8 (2.8)	−5.0^*∗∗*^	0.000
*Functional autonomy *				
ACDS-ADL inventory (/45)	37.6 (5.6)	41.9 (3.4)	−3.6^*∗∗*^	0.001
*Executive functioning *				
Letter-number sequencing (WAIS-IV; scaled score)	8.4 (2.0)	9.7 (1.6)	−2.6^*∗*^	0.013
D-KEFS Color-Word Interference Test Condition 3 (sec)	86.9 (26.7)	64.3 (12.2)	4.1^*∗∗*^	0.000
D-KEFS Color-Word Interference Test Condition 3 (errors)	3.0 (2.7)	1.1 (1.6)	3.2^*∗*^	0.003
D-KEFS Color-Word Interference Test Condition 4 (sec)	105.6 (39.4)	72.4 (21.2)	4.0^*∗∗*^	0.000
D-KEFS Color-Word Interference Test Condition 4 (errors)	5.3 (5.0)	1.9 (1.6)	3.6^*∗*^	0.001
D-KEFS Trail Making Test Condition 4 (sec)	156.4 (70.3)	109.6 (33.6)	3.2^*∗*^	0.002
D-KEFS Trail Making Test Condition 4 (errors)	0.9 (2.9)	0.0 (0.2)	1.6	0.129
*Visuoconstruction *				
Copy of Rey Figure (/36)	28.9 (4.9)	31.9 (3.2)	−2.7^*∗*^	0.009
*Language *				
Letter (T-N-P) fluency (number of words in 1 min)	29.2 (9.0)	35.4 (10.1)	−2.5^*∗*^	0.015
Semantic fluency (number of words in 1 min)	14.7 (4.9)	17.3 (4.4)	−2.2	0.033
Boston Naming Test (/15)	12.6 (1.7)	13.4 (1.3)	−2.2	0.033
*Semantic memory *				
Pyramids and Palm Trees Test (/52)	48.5 (3.2)	49.9 (1.8)	−2.1	0.041
*Episodic memory *				
Recall of the Rey Figure (3 min; /36)	12.1 (5.8)	17.6 (6.0)	−3.6^*∗∗*^	0.001
RL-RI free recall (/16)^b^	6.0 (1.8)	9.8 (1.2)	−9.7^*∗∗*^	0.000
RL-RI total recall (/16)^c^	12.2 (2.5)	15.5 (0.7)	−7.1^*∗∗*^	0.000
RL-RI delayed free recall (/16)	6.6 (3.7)	12.6 (2.1)	−7.8^*∗∗*^	0.000
RL-RI total delayed total recall (/16)	12.7 (2.9)	15.7 (0.4)	−5.8^*∗∗*^	0.000

*Note*. ACDS-ADL = Alzheimer's Disease Cooperative Study-Activities of Daily Living; D-KEFS = Delis-Kaplan Executive Function System; GDS = Geriatric Depression Scale; HC = healthy control participants; MCI = elderly person with mild cognitive impairment; MoCA = Montreal Cognitive Assessment; PPTT = Pyramid and Palm Trees Test; QPC = questionnaire de plainte cognitive; RL/RI-16 = Épreuve de rappel libre/rappel indicé à 16 items; SD = standard deviation; WAIS = Wechsler Adult Intelligence Scale.

^*∗*^ *p* < 0.05.

^*∗∗*^ *p* < 0.001 compared to control participants.

^a^Statistical differences for distribution of gender in each group were examined using Pearson Chi-Square test.

^b^This score was calculated as the mean number of words retrieved over the three free recall trials.

^c^This score was calculated as the mean total number of words retrieved on all free plus cued recall trials.

## References

[1] Albert M. S., DeKosky S. T., Dickson D. (2011). The diagnosis of mild cognitive impairment due to Alzheimer's disease: recommendations from the National Institute on Aging-Alzheimer's Association workgroups on diagnostic guidelines for Alzheimer's disease. *Alzheimer's & Dementia*.

[2] Gauthier S., Reisberg B., Zaudig M. (2006). Mild cognitive impairment. *The Lancet*.

[3] Baglio F., Castelli I., Alberoni M. (2012). Theory of mind in amnestic mild cognitive impairment: an fMRI study. *Journal of Alzheimer's Disease*.

[4] Castelli I., Baglio F., Blasi V. (2010). Effects of aging on mindreading ability through the eyes: an fMRI study. *Neuropsychologia*.

[5] Kemp J., Després O., Sellal F., Dufour A. (2012). Theory of Mind in normal ageing and neurodegenerative pathologies. *Ageing Research Reviews*.

[6] Frith U., Frith C. D. (2003). Development and neurophysiology of mentalizing. *Philosophical Transactions of the Royal Society B: Biological Sciences*.

[7] Saxe R., Baron-Cohen S. (2007). *Theory of Mind: A Special Issue of Social Neuroscience*.

[8] Champagne-Lavau M., Joanette Y. (2009). Pragmatics, theory of mind and executive functions after a right-hemisphere lesion: different patterns of deficits. *Journal of Neurolinguistics*.

[9] Channon S., Pellijeff A., Rule A. (2005). Social cognition after head injury: sarcasm and theory of mind. *Brain and Language*.

[10] Freedman M., Stuss D. T. (2011). Theory of Mind in Parkinson's disease. *Journal of the Neurological Sciences*.

[11] Monetta L., Grindrod C. M., Pell M. D. (2009). Irony comprehension and theory of mind deficits in patients with Parkinson's disease. *Cortex*.

[12] Spotorno N., Koun E., Prado J., Van Der Henst J.-B., Noveck I. A. (2012). Neural evidence that utterance-processing entails mentalizing: the case of irony. *NeuroImage*.

[13] Gaudreau G., Hudon C., Monetta L. (2011). Bases psycholinguistiques et neuroanatomiques de la compréhension de l'ironie chez l'adulte. *Revue de Neuropsychologie*.

[14] Gaudreau G., Monetta L., Macoir J., Laforce R., Poulin S., Hudon C. (2013). Verbal irony comprehension in older adults with amnestic mild cognitive impairment. *Neuropsychology*.

[15] Maki Y., Yamaguchi T., Koeda T., Yamaguchi H. (2013). Communicative competence in Alzheimer's disease: metaphor and sarcasm comprehension. *American Journal of Alzheimer's Disease and other Dementias*.

[16] Poletti M., Bonuccelli U. (2013). Alteration of affective Theory of Mind in amnestic mild cognitive impairment. *Journal of Neuropsychology*.

[17] Bull R., Phillips L. H., Conway C. A. (2008). The role of control functions in mentalizing: dual-task studies of Theory of Mind and executive function. *Cognition*.

[18] Dennis M., Agostino A., Roncadin C., Levin H. (2009). Theory of mind depends on domain-general executive functions of working memory and cognitive inhibition in children with traumatic brain injury. *Journal of Clinical and Experimental Neuropsychology*.

[19] Moreau N., Viallet F., Champagne-Lavau M. (2013). Using memories to understand others: the role of episodic memory in theory of mind impairment in alzheimer disease. *Ageing Research Reviews*.

[20] Spreng R. N., Mar R. A. (2012). I remember you: a role for memory in social cognition and the functional neuroanatomy of their interaction. *Brain Research*.

[21] Uekermann J., Channon S., Daum I. (2006). Humor processing, mentalizing, and executive function in normal aging. *Journal of the International Neuropsychological Society*.

[22] American Psychiatric Association (2003). *Diagnostic and Statistical Manual of Mental Disorders*.

[23] Yesavage J. A., Brink T. L., Rose T. L. (1982). Development and validation of a geriatric depression screening scale: a preliminary report. *Journal of Psychiatric Research*.

[24] Galasko D., Bennett D. A., Sano M., Marson D., Kaye J., Edland S. D. (2006). ADCS prevention instrument project: assessment of instrumental activities of daily living for community-dwelling elderly individuals in dementia prevention clinical trials. *Alzheimer Disease & Associated Disorders*.

[25] Anterion C. T., Ribas C., Honoré-Masson S., Berne G., Ruel J. H., Laurent B. (2003). Le questionnaire de plainte cognitive (QPC): un outil de recherche de plainte suspecte d'évoquer une maladie d'Alzheimer? [Cognitive Complaint Questionnaire]. *L'Année Gérontologique*.

[26] Jurica P. J., Leitten C. L., Mattis S. (2001). *Dementia Rating Scale-2: Professional Manual*.

[27] Nasreddine Z. S., Chertkow H., Phillips N., Whitehead V., Collin I., Cummings J. L. (2004). The Montreal Cognitive Assessment (MoCA): a brief cognitive screening tool for detection of mild cognitive impairment. *Neurology*.

[28] Delis D. C., Kaplan E., Kramer J. H. (2001). *The Delis-Kaplan Executive Function System: Examiner's Manual*.

[29] Wechsler D. (2008). *Wechsler Adult Intelligence Scale—Fourth Edition*.

[30] Rey A. (1960). *Test de la Figure Complexe de Rey*.

[31] Riddoch M. J., Humphreys G. W. (1993). *Birmingham Object Recognition Battery*.

[32] Mack W. J., Freed D. M., Williams B. W., Henderson V. W. (1992). Boston naming test: shortened versions for use in Alzheimer's disease. *Journals of Gerontology*.

[33] Consortium des Universités de Montréal et McGill (1996). *Manuel de l'Examen Neuropsychologique*.

[34] Howard D., Patterson K. (1992). *The Pyramids and Palm Trees Test*.

[35] Van der Linden M., Coyette F., Pointrenaud J., Gremem M. O. T., Van der Linden M., Adam S., Agniel A., Gremem M. O. T. (2004). L'épreuve de rappel libre/rappel indicé è 16 items (RL/RI-16) [16 items free recall/cued recall test]. *L'évaluation des troubles de la mémoire: présentation de quatre tests de mémoire épisodique (avec leur étalonnage)*.

[36] Achim A. M., Ouellet R., Roy M.-A., Jackson P. L. (2012). Mentalizing in first-episode psychosis. *Psychiatry Research*.

[37] Eviatar Z., Just M. A. (2006). Brain correlates of discourse processing: an fMRI investigation of irony and conventional metaphor comprehension. *Neuropsychologia*.

[38] Barbin M.-C., Robichaud D. (2010). *Création d'une tâche visant l'évaluation de la compréhension des ironies en contexte d'IRMf s'adressant á une population franco-canadienne [Mémoire de thèse dirigé par Dr Laura Monetta et Dr Thomas Jubault]*.

[39] Champagne-Lavau M., Stip E. (2010). Pragmatic and executive dysfunction in schizophrenia. *Journal of Neurolinguistics*.

[40] Martin I., McDonald S. (2004). An exploration of causes of non-literal language problems in individuals with Asperger Syndrome. *Journal of Autism and Developmental Disorders*.

[41] Aboulafia-Brakha T., Christe B., Martory M.-D., Annoni J.-M. (2011). Theory of mind tasks and executive functions: a systematic review of group studies in neurology. *Journal of Neuropsychology*.

[42] Charlton R. A., Barrick T. R., Markus H. S., Morris R. G. (2009). Theory of mind associations with other cognitive functions and brain imaging in normal aging. *Psychology and Aging*.

[43] Muller F., Simion A., Reviriego E. (2010). Exploring theory of mind after severe traumatic brain injury. *Cortex*.

[44] Saltzman J., Strauss E., Hunter M., Archibald S. (2000). Theory of mind and executive functions in normal human aging and Parkinson's disease. *Journal of the International Neuropsychological Society*.

[45] Shamay-Tsoory S. G., Tomer R., Berger B. D., Goldsher D., Aharon-Peretz J. (2005). Impaired ‘affective theory of mind’ is associated with right ventromedial prefrontal damage. *Cognitive and Behavioral Neurology*.

[46] Cheang H. S., Pell M. D. (2008). The sound of sarcasm. *Speech Communication*.

[47] Borella E., Ghisletta P., de Ribaupierre A. (2011). Age differences in text processing: the role of working memory, inhibition, and processing speed. *Journals of Gerontology—Seried B: Psychological Sciences and Social Sciences*.

[48] McGinnis D. (2009). Text comprehension products and processes in young, young-old, and old-old adults. *Journals of Gerontology—Series B: Psychological Sciences and Social Sciences*.

[49] Moran J. M. (2013). Lifespan development: the effects of typical aging on theory of mind. *Behavioural Brain Research*.

[50] Fjell A. M., Walhovd K. B. (2010). Structural brain changes in aging: courses, causes and cognitive consequences. *Reviews in the Neurosciences*.

[51] Duval C., Piolino P., Bejanin A., Eustache F., Desgranges B. (2011). Age effects on different components of theory of mind. *Consciousness and Cognition*.

[52] Li X., Wang K., Wang F., Tao Q., Xie Y., Cheng Q. (2013). Aging of theory of mind: the influence of educational level and cognitive processing. *International Journal of Psychology*.

